# Supplementary material for: Providing context: Extracting non-linear and dynamic temporal motifs from brain activity
Source: PLoS One. 2025 Jun 12;20(6):e0324066. doi: 10.1371/journal.pone.0324066 (PMC12161560; doi:10.1371/journal.pone.0324066)
Supplement: S1 Appendix — (ZIP) [file pone.0324066.s001.zip › S4_Appendix.pdf]

S4 Appendix: Convolutional vs RNN context encoder

To understand the effect of a different neural network architecture on the performance of our model's, we compare using a 1D CNN as the context embedding network to the original GRU. In Fig 1 we show the differences in classification accuracy between the original DSVAE/IDSVAE models, and DSVAE/IDSVAE models with a 1D CNN encoder. We use a 2D local size for our comparisons, and vary the context size for both model types.

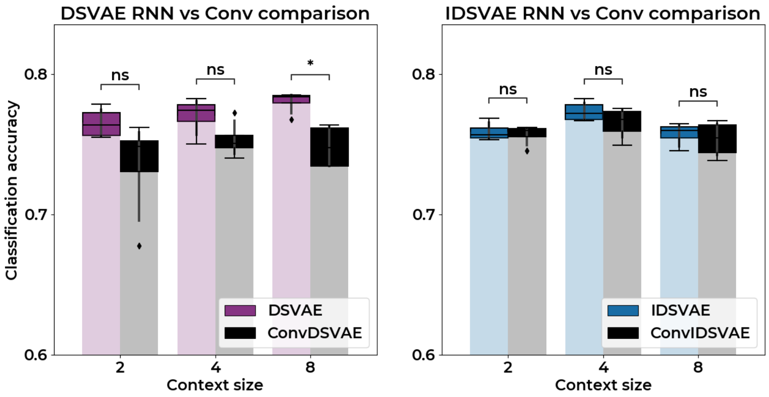

Figure 1: A comparison between using a GRU and a 1D-convolutional network as the context encoder. All models have a local size of 2, and the GRU-based networks generally outperform the convolution-based networks. The GRU-based DSVAE model with an 8-dimensional context size significantly outperforms the convolution-based model ($p < 0.05$).

The GRU-based DSVAE/IDSVAE models generally outperform the convolution-based versions in Fig 1, and the 8-dimensional GRU-based DSVAE model even significantly outperforms the convolution-based model ($p < 0.05$). The GRU-based context embedding network is thus generally preferable to the convolution-based one in our experiments.
