# Supplementary material for: Providing context: Extracting non-linear and dynamic temporal motifs from brain activity
Source: PLoS One. 2025 Jun 12;20(6):e0324066. doi: 10.1371/journal.pone.0324066 (PMC12161560; doi:10.1371/journal.pone.0324066)
Supplement: S1 Appendix — (ZIP) [file pone.0324066.s001.zip › S1_Appendix.pdf]

S1 Appendix: Hyperparameter ranges

To select the best hyperparameter combination, we train each hyperparameter combination with 4 different randomly initialized networks. After training all the hyperparameter combinations, we average their performance across these four random initializations, and select the model with the best average validation loss. The hyperparameter ranges for the DSVAE and IDSVAE models are presented in Table 1. The 'number of layers' hyperparameter determines the number of hidden layers in the MLP we use as a decoder. Thus, 0 layers would indicate a completely linear mapping between the latent space and the original data space. The 'spatial hidden size' hyperparameter determines the size of the hidden size of the hidden layer in the MLP we use as a decoder, and it is also used for other MLPs in the model. The 'temporal hidden size' hyperparameter determines the hidden size in the GRU(s) or the last layer of the CNN. Dropout is applied after the hidden layers of an MLP, in the CNN, and after GRUs. Moreover, all models are trained with the Adam optimizer [1], a 0.002 learning rate, and $1E-5$ weight decay for 1000 epochs, with a 50 epoch early stopping criterion.

Hyperparameter	Number of layers	Spatial hidden size	Temporal hidden size	Dropout
Range	[0, 1, 2, 3]	[128, 256]	[256, 512]	[0.05, 0.1]

Table 1: The hyperparameter ranges that were optimized over.

References

[1] Kingma DP. Adam: A method for stochastic optimization. arXiv preprint arXiv:14126980. 2014;.
