# Supplementary material for: Providing context: Extracting non-linear and dynamic temporal motifs from brain activity
Source: PLoS One. 2025 Jun 12;20(6):e0324066. doi: 10.1371/journal.pone.0324066 (PMC12161560; doi:10.1371/journal.pone.0324066)
Supplement: S1 Appendix — (ZIP) [file pone.0324066.s001.zip › S2_Appendix.pdf]

S2 Appendix: The influence of training-time noise on classification accuracy and reliability

To quantify the effect of training-time noise on the classification accuracy and reliability of our models, we trained three DSVAE and three IDSVAE models with additive training-time Gaussian noise. We used LS=2,CS=2 as the model settings for each, and used three noise levels: 0.01 standard deviation (low), 0.05 standard deviation (medium), and 0.1 standard deviation (high). The noise was added indendently to each sample during batch generation. An example's added noise thus varies across epochs. Then, we ran the same classification accuracy experiment as in Section Window Classification on data without any noise. The results are shown in Fig 1.

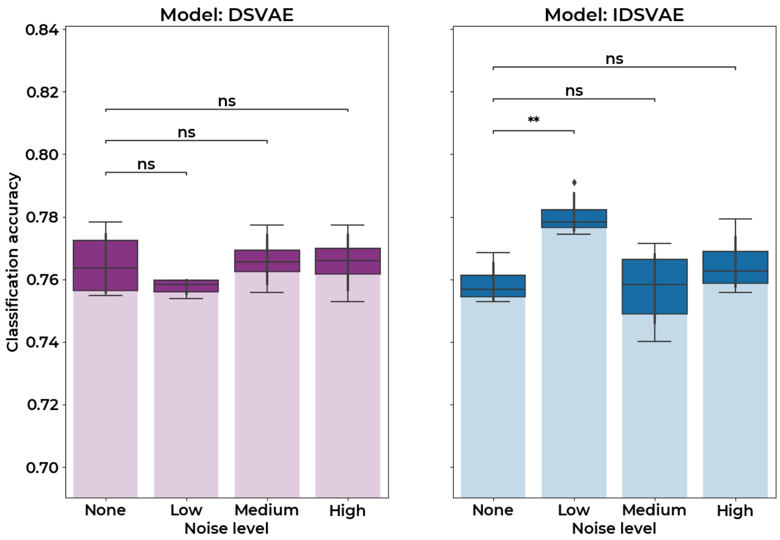

Figure 1: Models trained with various levels of training-time noise. During inference, each model uses the noiseless version of the data. Training-time noise does not seem to affect the models much, regardless of the strength of the noise, in fact, for the IDSVAE model, a low level of added training-time noise significantly improves classification accuracy ($p < 0.01$).

Training-time noise does not have a significant effect on the DSVAE model, regardless of the noise level. In all but one case, training-time noise does not have a significant effect on the IDSVAE model's classification accuracy either. When we add a low level of training-time noise, the IDSVAE model's classification accuracy significantly increases ($p < 0.01$). This indicates that training-time noise does not hurt our models' classification accuracy.

To test how reliable the model remains with training-time noise, we also test how similar the embedding space for each noise level is when compared to embedding space of the model without training-time noise. This analysis is similar to the experiment in Section Reliability Analysis, but we now compare the embedding space of a model trained with a certain noise level to the embedding space of the same model and seed without the training-time noise. The similarity measure is averaged across the four seeds. This measures how similar the embedding space remains even with training-time noise added to the model. The results are shown in Fig 2.

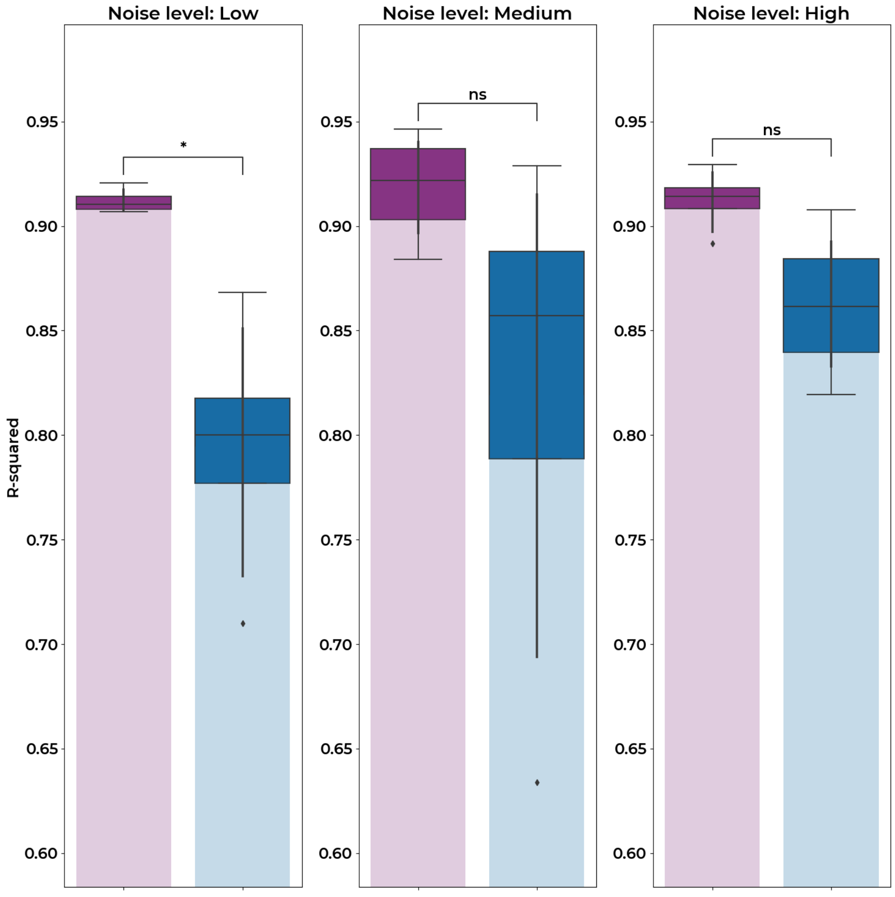

Figure 2: The reliability of each model across three different noise levels. The reliability is measured based on the linear similarity between a model with no training-time noise, and a model trained with a certain level of training-time noise. Generally, the DSVAE model performs better than the IDSVAE model, and significantly better for a low noise level ($p < 0.05$).

The results in Fig 2 indicate that the DSVAE model is generally more robust to noise in terms of learning a similar embedding space. Specifically for the low noise level, the DSVAE model is significantly more reliable ($p < 0.05$) than the IDSVAE model. It is important to note however that this IDSVAE model obtains a significantly higher classification accuracy as well. The significantly higher classification accuracy indicates a different embedding space arrangement that separates subjects diagnosed with schizophrenia and control subjects better. Hence, the embedding space is not as similar to the IDSVAE model trained without training-time noise.
