# Supplementary material for: Providing context: Extracting non-linear and dynamic temporal motifs from brain activity
Source: PLoS One. 2025 Jun 12;20(6):e0324066. doi: 10.1371/journal.pone.0324066 (PMC12161560; doi:10.1371/journal.pone.0324066)
Supplement: S1 Appendix — (ZIP) [file pone.0324066.s001.zip › S3_Appendix.pdf]

S4 Appendix: Geometric manifold comparison visualization

To visually verify that our model's embedding space is not capturing wFNC features, we visualize our model's embedding space as a grid using a modified Jonker-Volgenant algorithm [1, 2] in Fig 1. The plot is created by first embedding each of the windows using our LS=4,CS=2 model, the same model we used in Section Cluster analysis. Then, we used the modified Jonker-Volgenant algorithm to map the embedded windows to points in a grid. Lastly, we visualized each point/window as its wFNC, this allows us to see whether wFNC patterns are close together in our model's embedding space. If this is not the case, then our model is likely capturing complementary features that are important (see Section Window classification), and interesting (see Section Cluster analysis). In Fig 1, we can see that similar wFNCs are not per se close together in our

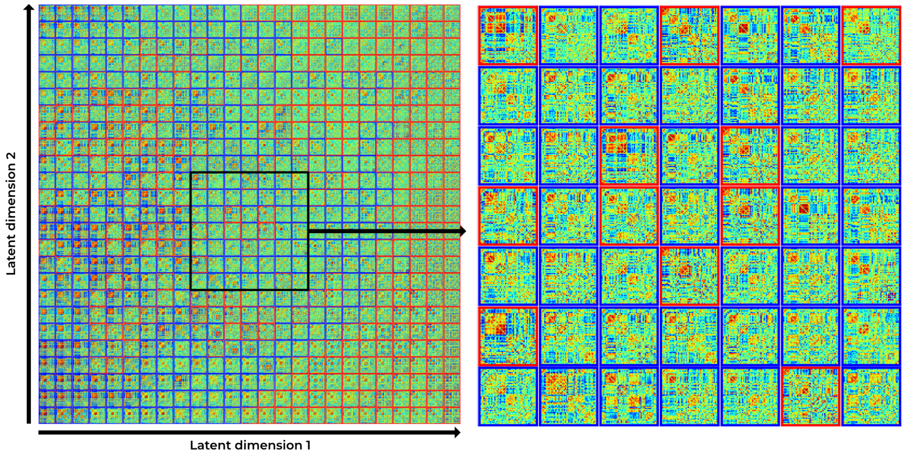

Figure 1: The left plot shows the points in our model's embedding space represented as wFNC matrices. Each wFNC's border is either red or blue; red corresponds to windows that are from schizophrenia patients and blue corresponds to windows from control subjects. The right side shows a zoomed-in version of the subplot on the left.

model's embedding space. Since correlation is invariant to scaling, temporal permutation (if the permutation is the same for all inputs), and the addition of a constant, connectivity features from correlations are highly specific. Indeed, we can visually see that our model captures different features than wFNC does. Thus, our method can uncover novel yet complementary motifs for this schizophrenia population from rs-fMRI data.

References

[1] Crouse DF. On implementing 2D rectangular assignment algorithms. IEEE Transactions on Aerospace and Electronic Systems. 2016;52(4):1679–1696.

[2] Virtanen P, Gommers R, Oliphant TE, Haberland M, Reddy T, Cournapeau D, et al. SciPy 1.0: fundamental algorithms for scientific computing in Python. Nature methods. 2020;17(3):261–272.
